# Stealth cryptococcus in an immunocompetent patient

**DOI:** 10.4322/acr.2024.520

**Published:** 2024-09-27

**Authors:** Emily Ryan, Gia Jackson, Larry Nichols

**Affiliations:** 1 Mercer University, School of Medicine, Department of Pathology and Clinical Science Education, Macon, GA, USA

**Keywords:** Cryptococcosis, Meningitis, Headache, Immunocompetence, Diagnosis, Differential

## Abstract

Cryptococcosis occurs primarily in immunocompromised patients. It is difficult to suspect in an immunocompetent patient presenting with a headache. The clinical manifestations of cryptococcosis can be subtle in a patient whose immune system is responding, but inadequate. This is the report of a case of fatal cryptococcosis initially misdiagnosed as a sinus headache on the basis of a telephone call, and then misdiagnosed as aseptic meningitis on the basis of mild findings and negative cerebrospinal fluid cultures. Autopsy revealed unsuspected severe cryptococcal meningoencephalitis. Cerebrospinal fluid nuclear acid amplification (NAA) panels including Cryptococcus should enable the diagnosis of unsuspected cryptococcal meningitis in most cases, but can be false positive, which could be adjudicated by cryptococcal antigen and culture. It will remain important to test for cryptococcal antigen and to maintain a broad differential diagnosis for all patients with meningitis.

## INTRODUCTION

It can be difficult to suspect a disease in a patient from a population in which the disease is rare. This becomes important if the disease is treatable. Cryptococcosis is a treatable disease that occurs primarily in immunocompromised patients.^1^ This case illustrates what can happen when cryptococcosis is not suspected in an immunocompetent patient. This case also illustrates the difficulty of diagnosing a rare cause of a very common symptom (cryptococcosis as a cause of headache) and the pitfalls of trying to diagnose the cause of a headache in a telephone consultation. This case has lessons for patient care.

## CASE REPORT

A 48-year-old man presented with a headache and nausea for 2 days. He was a truck driver and lived in a rural area in the Mideast United States. He developed a headache and also felt “sick to his stomach” upon returning home from a trip to the Northeast United States in September. He called his primary care physician two days later and was prescribed a course of azithromycin for presumptive sinus infection. The patient had a history of diabetes mellitus, hypertension, and hyperlipidemia. He was of European ancestry and had history of smoking 3 packs (60 cigarettes) per day for 20 years. After starting azithromycin, the patient felt a little better the next day, but his wife noted that he was slurring words when speaking. He continued to have a pounding headache and feeling poorly, which prompted admission to the small rural hospital in his community on day 5 of his illness.

In the small rural community hospital, a lumbar puncture was performed and the cerebrospinal fluid (CSF) showed 840 white blood cells/μL (98% lymphocytes), protein 119 mg/dL, and glucose 71 mg/dL. Empirical antibiotic and steroid therapy were given. The patient felt better the next day. CSF cultures were negative. The patient’s symptoms were felt to be due to aseptic meningitis and empirical antibiotic therapy was discontinued on his third day in the hospital. The following day, the patient’s condition deteriorated. He became very agitated, and was transferred to a regional secondary care hospital on his fourth day in hospital.

In the secondary care hospital, another lumbar puncture was performed and the CSF showed 1453 white blood cells/μL (44% lymphocytes, 41% neutrophils), protein 150 mg/dL, and glucose 42 mg/dL. Empirical antibiotic therapy was given. Cerebrospinal fluid cultures and polymerase chain reaction tests for herpes simplex virus and eastern equine encephalitis virus were negative. The patient failed to improve and he was transferred to a large urban tertiary care referral hospital on his second day in the second hospital.

In the tertiary care referral hospital, the patient was treated with empirical ceftriaxone, trimethoprim-sulfamethoxazole and vancomycin. On his second day in the referral hospital, magnetic resonance imaging (MRI) showed scattered areas of focal signal changes and restricted diffusion, mainly cortically based in the medial aspect of the right frontal lobe and left superior frontal gyrus as well as right basal ganglia and right thalamus. Magnetic resonance angiography (MRA) showed approximately 20% stenosis of the left internal carotid artery, diffuse attenuation of the basilar artery with no focal stenoses, patent posterior cerebral arteries, unremarkable anterior intracranial circulation, and patent dural sinuses. The patient required intubation and mechanical ventilation; he became hypotensive and required vasopressor therapy.

On the third day in the referral hospital, the patient was febrile and sedated, not following commands. Sedation was held and he remained unresponsive. On hospital day 4 in the referral hospital, computed tomography (CT) of the head showed diffuse edema, and bilateral infarction in anterior cerebral artery territories. Electroencephalogram showed grade III generalized suppression. After 24 hours off sedation, electroencephalogram showed a flat line consistent with brain death. On hospital day 5 in the referral hospital, the patient's white blood cell (WBC) count was 20,000/mm^3^ (RR: 3,800-10,600/mm^3^) [79% neutrophils, 9% lymphocytes, 10% monocytes], platelets 620,000/mm^3^ (RR: 156,000-369,000/mm^3^), international normalized ratio (INR) 1.1 (RR: 0.8-1.2), partial thromboplastin time 33.7 seconds (RR: 25-33 seconds), blood urea nitrogen (BUN) 10 mg/dL (RR: 5-20 mg/dL), creatinine 1.1 mg/dL (RR: 0.5-1.4 mg/dL), creatine phosphokinase (CPK) 448 U/L (RR: 25-90 U/L), albumin 3.3 g/dL (RR: 3.5-5.0 g/dL), bilirubin 0.2 mg/dL (RR: 0.1-1.0 mg/dL), alkaline phosphatase 91 U/L (RR: 40-125 U/L), alanine aminotransferase (ALT) 23 U/L (RR: <40 U/L), and aspartate aminotransferase (AST) 32 U/L (RR: <40 U/L). Arterial blood showed pH 7.28 (RR: 7.35-7.45), PCO2 46 mm Hg (RR: 35-45 mm Hg), and PO2 66 mm Hg (RR: 60-100 mm Hg), on mechanical ventilation with 100% oxygen. The patient was a candidate for organ donation and evaluation for that purpose included serology for human immunodeficiency virus (HIV), syphilis and hepatitis viruses, which was negative. A decision was reached with his family to switch to comfort measures only and he died 14 days after the onset of his illness.

## AUTOPSY FINDINGS

**Figure 1:**
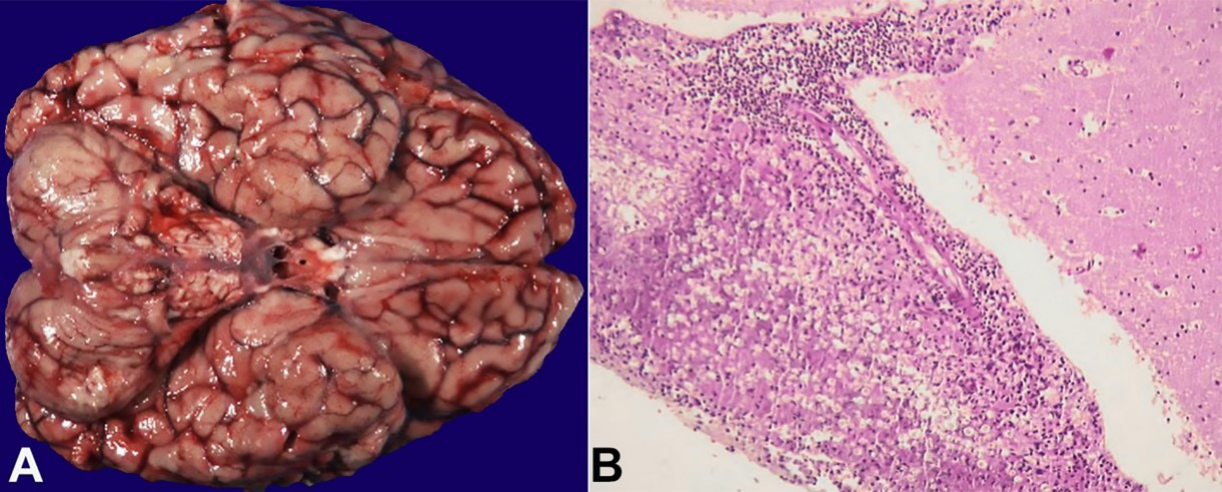
**A –** gross view of the brain showing a smooth and glistening inferior surface of the brain; **B –** photomicrograph of the brain with lymphocytic meningitis over the cerebellum (H&E, 40X).

Autopsy revealed diffuse severe cryptococcal meningitis of the brain and spinal cord, with infection focally extending into the brain, and global cerebral hypoxic-ischemic changes. On gross examination, the surface of the brain was deceptively unremarkable (Figure 1A). Microscopic examination revealed meningitis with fibrinous exudate, debris and leukocytes, predominantly lymphocytes (Figure 1B).

**Figure 2:**
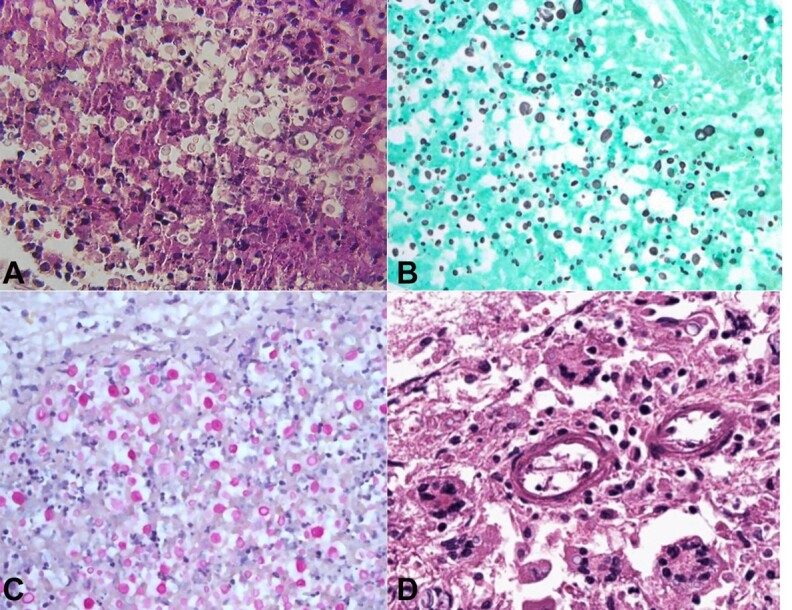
Photomicrographs of the brain. **A –** Variably sized yeast forms with surrounding cleared spaces (H&E, 400X); **B –** Numerous yeast forms of various sizes (GMS, 400X); **C –** Positively stained fungal capsules within cleared spaces (Mucicarmine, 400X); **D –** Multinucleated giant cells, some with a Langhans type peripheral arrangement of nuclei and some containing yeast forms (H&E, 200X).

Under higher magnification, within the exudate, numerous variably sized, lightly stained, amphophilic yeast forms with cleared spaces around them, a few of the yeast budding, were evident (Figure 2A). Grocott methenamine silver stain highlighted the yeast forms (Figure 2B). Mucicarmine stain showed pink positive staining of fungal capsules (Figure 2C). Unlike rapidly fatal cryptococcal infection in immunocompromised patients, in this case, there was early granulomatous inflammation in the meninges (Figure 2D).

Autopsy also revealed focal pulmonary cryptococcosis in subpleural right upper lobe lung, along with mild pulmonary emphysema, mild respiratory bronchiolitis, moderate muscular wall thickening of pulmonary arteries, and rare tiny microscopic pulmonary thromboemboli. The coronary arteries showed severe atherosclerosis, with up to approximately 85% stenosis of the right coronary artery, and up to about 75% stenosis of the left anterior descending and circumflex coronary arteries. Postmortem cultures of blood and randomly selected lung tissue were negative.

## DISCUSSION

Cryptococcal meningitis is uniformly fatal without antifungal treatment.^1^ This makes cryptococcal meningitis an important diagnosis not to miss. There are estimated to be 223,000 new cases of cryptococcosis per year worldwide, with over 180,000 deaths per year.^2^ The incidence of cryptococcal meningitis in Europe and the Americas has doubled since 2009, while remaining stable in other parts of the world.^3^ There is a wide spectrum of patients with cryptococcosis, from immunocompetent hosts without an underlying disease to those severely immunocompromised from human immunodeficiency virus infection, immunosuppression for transplantation, or malignancy. The majority of patients with cryptococcosis are immunocompromised. This makes it difficult to suspect cryptococcosis in an immunocompetent patient.

Immunocompetent patients tend to get infection with Cryptococcus gatti rather than Cryptococcus neoformans.^2^ Cryptococcus neoformans has a worldwide distribution, but Cryptococcus gatti has been mainly in places with a tropical or subtropical climate. Cryptococcus gatti infection in apparently immunocompetent patients has been linked to subtle immunodeficiency such as compromised pulmonary alveolar macrophage function associated with autoantibodies against granulocyte-macrophage colony-stimulating factor.^2^ Immunocompetent patients with cryptococcosis may experience clinical deterioration after biologic control (negative cultures) during effective fungicidal therapy, apparently due to the release of fungal products; this is termed a postinfectious inflammatory response syndrome.^2^ In the case of this report, the patient received no antifungal therapy, so his clinical deterioration cannot have been due to a postinfectious inflammatory response syndrome. The case of this report occurred in a region at the northern limit of a temperate climate, transitional between the continental and subtropical climate zones. It is possible the infection in this case was due to Cryptococcus gatti, but no cultures were positive to allow identification of the species.

Diagnostic methods for detecting cryptococcus in CSF range from the historic India ink method to molecular nucleic acid amplification (NAA) methods, and they vary in their analytical target and their efficiency in clinical use. The common methods used are culture, India ink microscopy, cryptococcal antigen, and molecular NAA tests. Culture and India ink microscopy require intact organisms within the CSF for detection. In culture on most routine laboratory agar media, colonies of Cryptococcus species appear within 48 to 72 hours after plating a specimen.^1^ India ink testing has fallen out of favor as it is the least sensitive method available.^4^ In immunocompetent individuals, cryptococcus organism burden tends to be low initially, and the few organisms present then may be "viable but not culturable", leading to false negative culture results.^3^ Due to the inherent delay in culture results, cryptococcal antigen testing has become popular for diagnosing symptomatic cases. Antigen testing can be used on multiple specimen types, but the most common are serum and CSF. Cryptococcal antigen testing is more sensitive than culture in both immunocompromised and immunocompetent populations.^4^ The first-generation type of cryptococcal antigen testing, using a latex agglutination method, has been surpassed by lateral flow assays, which are much more sensitive and can be used (via titers) to monitor treatment response. Lateral flow assays are now used to screen patients in resource-limited locations where the more expensive molecular NAA panels or, the more laborious culture may not be available.^3^

CSF meningitis molecular NAA panels that amplify DNA or RNA from multiple bacterial, viral, and fungal targets have become common in resource-abundant settings, and allow specific viral etiologies of meningitis to be diagnosed in cases previously diagnosed as aseptic meningitis. For cryptococcal meningitis, NAA panel results have also been shown to correlate more closely with antigen testing rather than the gold standard culture.^5^ Either an antigen assay or NAA testing can detect cryptococcal infection, but antigen assay titers can also be used to monitor the effectiveness of treatment. Cerebrospinal fluid NAA panel testing can be false-positive for cryptococcus, and this can be adjudicated by antigen testing and culture.^5^

In this case, the CSF initially showed a lymphocytic pleocytosis. Follow-up CSF showed increasing pleocytosis, partly neutrophilic, associated with rising CSF protein and falling CSF glucose consistent with bacterial or fungal meningitis masked by false negative CSF cultures. Acid-fast stain of CSF was not done; the incidence of mycobacterial meningitis in the region where this case occurred is extremely low. CSF NAA panels were not available at the time of this case; cryptococcal antigen testing was not done because cryptococcosis was not suspected. One can take multiple lessons from this case.

Perhaps the first lesson from this case is to keep cryptococcal meningitis in the differential diagnosis for headache in an immunocompetent patient. This patient lived in a rural area, and his initial hospitalizations were in rural areas. Clinicians in rural areas with few immunocompromised patients may lack experience with cryptococcosis, making it more difficult to suspect this disease in ambulatory patients presenting with a headache. Headache is among the most common reasons patients present for care in an ambulatory setting, and 90% are primary headaches that are usually not serious.^6^ This makes it hard to suspect cryptococcal meningitis in an immunocompetent patient presenting with a headache in an ambulatory care setting, but this fatal case illustrates the importance of doing so.

The second lesson from this case could be the importance of maintaining a broad differential diagnosis, including rare etiologies, for severe headaches. This applies in formulating the initial working diagnosis, avoiding the error of premature closure, and throughout patient management, avoiding anchoring error when evidence emerges that the initial working diagnosis is not correct. In this case, the first lumbar puncture yielded CSF with findings supporting a diagnosis of aseptic, presumptively benign viral meningitis, but the second lumbar puncture did not. In this case, however, the first lumbar puncture was at one hospital and the second at a different hospital. The patient was then soon transferred to a third hospital, where he rapidly went into shock with respiratory failure. With such a rapid clinical deterioration and the two hospital transfers, anchoring error was unlikely a factor in this case, but it is always important to remember and avoid.

In this case, cryptococcal meningitis was initially misdiagnosed as a sinus headache on the basis of a telephone call. Sinusitis is one of the most common conditions for which patients seek medical attention.^7^ In this instance, physical examination might have failed to show signs of sinusitis and may have shown early subtle signs of meningeal inflammation. The third lesson from this case could be to have a very low threshold for asking a patient presenting with a headache to be seen in person.

CSF testing using NAA panels, including cryptococcus, should enable the diagnosis of unsuspected cryptococcal meningitis in a case such as this one, but might require repeat lumbar puncture to obtain a cryptococcal antigen titer for the purpose of assessing the efficacy of therapy, unless the unexpected positive NAA test result was followed by a rapid clinician order for the test or laboratory reflex test for it. Perhaps the fourth lesson from this case is to test CSF for cryptococcal antigen in all cases of meningitis to obtain a titer, even in cases where cryptococcosis is not suspected.

## CONCLUSION

The first lesson from this case is to keep cryptococcal meningitis in the differential diagnosis for headache in an immunocompetent patient. The second lesson from this case might be the importance of maintaining a broad differential diagnosis for severe headaches, including rare etiologies. The third lesson from this case could be to have a very low threshold for asking a patient presenting with a headache to be seen in person. The fourth lesson is to test cerebrospinal fluid for cryptococcal antigen in all cases of meningitis to obtain a titer, even in cases where cryptococcosis is unsuspected.
